# Poly[[diaqua(μ_2_-4,4′-dipyridyl sulfide-κ^2^ *N*:*N*′)(4,4′-dipyridyl sulfide-κ*N*)(2-hydroxy-5-sulfonatobenzoato-κ*O* ^1^)nickel(II)] dihydrate]

**DOI:** 10.1107/S1600536808029206

**Published:** 2008-09-20

**Authors:** Zhong-Xiang Du, Jun-Xia Li

**Affiliations:** a Department of Chemistry and Chemical Engineering, Luoyang Normal University, Luoyang, Henan 471022, People’s Republic of China

## Abstract

The asymmetric unit of the title helical coordination polymer, {[Ni(C_7_H_4_O_6_S)(C_10_H_8_N_2_S)_2_(H_2_O)_2_]·2H_2_O}_*n*_, is comprised of an Ni^II^ ion, one 5-sulfosalicylic acid dianion (HSSA), two 4,4′-dipyridylsulfide (4,4′-dps) ligands, and two coordinated and two uncoordinated water mol­ecules. The Ni^II^ ion is coordinated by two water mol­ecules, one carboxyl­ate O atom of the HSSA dianion and three N atoms from three 4,4′-dps ligands in a distorted octa­hedral environment. Half of the 4,4′-dps ligands are μ_2_-bridging ligands which link adjacent Ni^II^ centers, forming a one-dimensional helical structure along the *b* axis. This helical structure is further stabilized by O—H⋯O intra- and inter­molecular hydrogen bonds.

## Related literature

For related structures, see: Fujita *et al.* (1994 ▶); Hao & Zhang (2007 ▶); Hou *et al.* (2001 ▶); Jung *et al.* (1999 ▶, 2000 ▶); Niu *et al.* (2006 ▶); Vaganova *et al.* (2004 ▶); Wen *et al.* (2004 ▶); Zeng *et al.* (2006 ▶); Zheng & Vittal (2001 ▶); Zheng *et al.* (1999 ▶). 
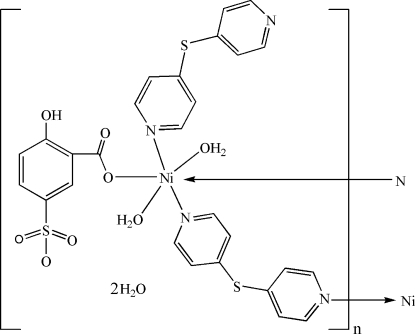


## Experimental

### 

#### Crystal data

[Ni(C_7_H_4_O_6_S)(C_10_H_8_N_2_S)_2_(H_2_O)_2_]·2H_2_O
*M* *_r_* = 723.42
Monoclinic, 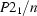

*a* = 11.4649 (10) Å
*b* = 13.9441 (12) Å
*c* = 20.7051 (18) Å
β = 96.5520 (10)°
*V* = 3288.5 (5) Å^3^
*Z* = 4
Mo *K*α radiation
μ = 0.84 mm^−1^
*T* = 291 (2) K
0.44 × 0.26 × 0.18 mm

#### Data collection

Bruker APEXII CCD area-detector diffractometer
Absorption correction: multi-scan (*SADABS*; Sheldrick, 1996 ▶) *T*_min_ = 0.709, *T*_max_ = 0.866
23823 measured reflections
6054 independent reflections
4328 reflections with *I* > 2σ(*I*)
*R*_int_ = 0.037

#### Refinement

*R*[*F* ^2^ > 2σ(*F* ^2^)] = 0.058
*wR*(*F* ^2^) = 0.178
*S* = 1.02
6054 reflections
426 parameters
219 restraints
H-atom parameters constrained
Δρ_max_ = 0.96 e Å^−3^
Δρ_min_ = −0.58 e Å^−3^

### 

Data collection: *APEX2* (Bruker, 2004 ▶); cell refinement: *APEX2*; data reduction: *SAINT* (Bruker, 2004 ▶); program(s) used to solve structure: *SHELXS97* (Sheldrick, 2008 ▶); program(s) used to refine structure: *SHELXL97* (Sheldrick, 2008 ▶); molecular graphics: *SHELXTL* (Sheldrick, 2008 ▶); software used to prepare material for publication: *SHELXTL*.

## Supplementary Material

Crystal structure: contains datablocks global, I. DOI: 10.1107/S1600536808029206/at2628sup1.cif

Structure factors: contains datablocks I. DOI: 10.1107/S1600536808029206/at2628Isup2.hkl

Additional supplementary materials:  crystallographic information; 3D view; checkCIF report

## Figures and Tables

**Table:** 

Ni1—O9	1.981 (3)
Ni1—N4^i^	2.040 (3)
Ni1—N1	2.041 (4)
Ni1—N3	2.055 (4)
Ni1—O2	2.430 (3)
Ni1—O5	2.437 (3)

**Table:** 

O9—Ni1—N4^i^	173.60 (14)
O9—Ni1—N1	88.00 (13)
O9—Ni1—N3	87.87 (13)
N1—Ni1—N3	175.23 (14)
O9—Ni1—O2	92.99 (12)
N1—Ni1—O2	87.06 (14)
O9—Ni1—O5	80.75 (12)
N1—Ni1—O5	93.08 (14)
O2—Ni1—O5	173.73 (12)

Symmetry code: (i) 
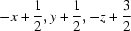
.

**Table 2:** Hydrogen-bond geometry (Å, °)

*D*—H⋯*A*	*D*—H	H⋯*A*	*D*⋯*A*	*D*—H⋯*A*
O11—H11⋯O10	0.82	1.84	2.535 (5)	142
O5—H10*W*⋯O3^ii^	0.83	1.98	2.797 (5)	170
O5—H9*W*⋯O4^iii^	0.85	2.04	2.721 (8)	136
O4—H7*W*⋯O8^iv^	0.83	2.30	2.713 (9)	112
O3—H6*W*⋯O6^v^	0.83	2.10	2.811 (7)	143
O3—H5*W*⋯O6^vi^	0.83	2.24	2.765 (8)	122
O2—H4*W*⋯O10	0.83	1.95	2.690 (5)	149
O2—H3*W*⋯O7^v^	0.84	1.87	2.652 (7)	155
O1—H1*W*⋯O11^vii^	0.85	2.03	2.876 (7)	180

Symmetry codes: (ii) 
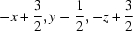
; (iii) 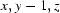; (iv) 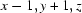; (v) 
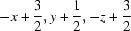
; (vi) 
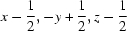
; (vii) 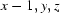.

**Fig. 1.**
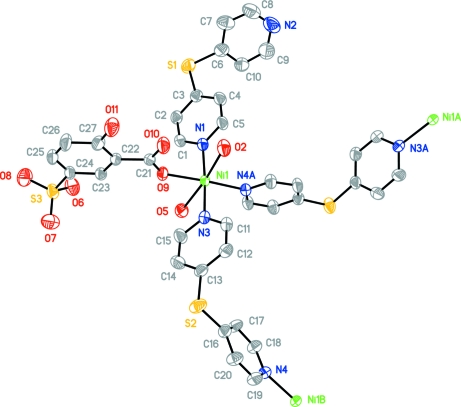
The coordination environment of NiII ion in (I), with displacement ellipsoids shown at the 30% probability level. Uncoordinate water molecules and H atoms have been omitted [Symmetry codes: (A) 1/2 - x, 1/2 + y, 3/2 - z; (B) 1/2 - x, -1/2 + y, 3/2 - z].

**Fig. 2.**
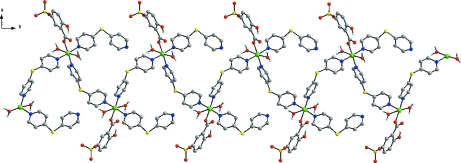
The helix structure for (I) along b axis. Uncoordinate water molecules and H atoms on C atoms have been omitted.

**Fig. 3.**
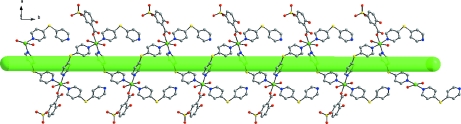
The helix structure for (I) along b axis with a helix axis. Uncoordinate water molecules and H atoms have been omitted.

**Fig. 4.**
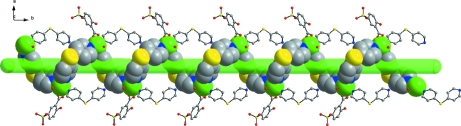
The space filled diagram of the helix structure for (I) along b axis. Uncoordinate water molecules and H atoms have been omitted.

## supplementary crystallographic information

### Comment

Recently, the use of bipyridyl based bridging ligands and transition metal centers in the preparation of various coordination compounds have attracted considerable interests not only because of their structural novelty but also for their potential properties in magnetism (Zheng *et al.*, 1999), nonlinear optics (Hou *et al.*, 2001), catalysts (Fujita *et al.*, 1994) and so on. 4,4'-dps possesses a magic angle (the angle of C—S—C almost equals to 100°) and conformational nonrigidity so it has some flexibility in contrast to linear rigid ligands such as simple 4,4'-bipyridine analogues. A number of metal complexes derived from 4,4'-dps have been reported previously. Among them, the 4,4'-dps has three kind of coordination modes and they are non-coordinate (Zeng *et al.*, 2006; Wen *et al.*, 2004; Vaganova *et al.*, 2004),µ_2_-bridging (Zheng & Vittal, 2001; Jung *et al.*, 2000; Hao & Zhang, 2007; Niu *et al.*, 2006), µ_2_ and µ_3_ together (Jung *et al.*, 1999). In this paper, we describe another new compound in which the 4,4'-dps is monodentate and µ_2_-bridging together, (I), (Fig. 1).

Complex (I) is composed of [Ni(C_10_H_8_N_2_S)_2_)(C_7_H_4_O_6_S)(H_2_O)_2_].2H_2_O units, in which the Ni^II^ ion is six-coordinated in a distorted octahedral geometry (Table 1) formed by two coordinate water molecules, one carboxylate O atom of HSSA dianion, one N atoms from monodentate 4,4'-dps ligand and another two N atoms from another two µ_2_-bridging 4,4'-dps ligands. Half of the 4,4'-dps are monodentate and the other half are µ_2_-bridging. It is just through the µ_2_-bridging function that the adjacent Ni^II^ centers are joined to form a one-dimensional helix structure (Fig. 2, 3 & 4) along *b* axis in the monoclinic unit cell, with the Ni···Ni(1/2 - *x*, 1/2 + *y*, 3/2 - *z*) distance of 10.6096 (10) Å. The phenolic hydroxyl and carboxyl of HSSA dianion are involved in intramolecular hydrogen bonding (Table 2). Together with the other O—H···O intermolecular hydrogen bonds with participation of water molecules, the helix structure are further stabilized.

### Experimental

The ligand 4,4'-dps (0.5 mmol, 0.14 g), 5-sulfosalicylic acid (0.5 mmol, 0.13 g) and NaOH (1.0 mmol, 0.04 g) were dissolved in water and methanol mixed solvent (30 ml, *v*/*v* 1:1). To this solution, Ni(CH_3_COO)_2_.4H_2_O (0.5 mmol, 0.13 g) was added and the resulting mixture was stirred and refluxed at 353 K for 2.5 h, then cooled to room temperature. After filtration and evaporation in air for 3 days, green block-shaped crystals were obtained in a yield of 32%. Analysis, found (%): C, 44.83; H 3.84, N 7.79, S13.22. C_27_H_28_N_4_NiO_10_S_3_ requires (%): C 44.78, H 3.87, N 7.74, S 13.27. [CCDC number 656224].

### Refinement

H atoms bonded to C atoms were positioned geometrically with C—H distance of 0.93 Å, and treated as riding atoms, with *U*_iso_(H) = 1.2*U*_eq_. H atoms bonded to O atoms were located in a difference Fourier map and refined isotropically.

### Crystal data

**Table:**

[Ni(C_7_H_4_O_6_S)(C_10_H_8_N_2_S)_2_(H_2_O)_2_]·2H_2_O	*F*(000) = 1496
*M**_r_* = 723.42	*D*_x_ = 1.461 Mg m^−^^3^
Monoclinic, *P*2_1_/*n*	Mo *K*α radiation, λ = 0.71073 Å
Hall symbol: -P 2yn	Cell parameters from 4865 reflections
*a* = 11.4649 (10) Å	θ = 2.3–21.6°
*b* = 13.9441 (12) Å	µ = 0.84 mm^−^^1^
*c* = 20.7051 (18) Å	*T* = 291 K
β = 96.552 (1)°	Block, green
*V* = 3288.5 (5) Å^3^	0.44 × 0.26 × 0.18 mm
*Z* = 4	

### Data collection

**Table:**

Bruker APEXII CCD area-detector diffractometer	6054 independent reflections
Radiation source: fine-focus sealed tube	4328 reflections with *I* > 2σ(*I*)
graphite	*R*_int_ = 0.037
φ and ω scans	θ_max_ = 25.5°, θ_min_ = 2.4°
Absorption correction: multi-scan (SADABS; Sheldrick, 1996)	*h* = −13→13
*T*_min_ = 0.709, *T*_max_ = 0.866	*k* = −16→16
23823 measured reflections	*l* = −25→25

### Refinement

**Table:**

Refinement on *F*^2^	Primary atom site location: structure-invariant direct methods
Least-squares matrix: full	Secondary atom site location: difference Fourier map
*R*[*F*^2^ > 2σ(*F*^2^)] = 0.058	Hydrogen site location: inferred from neighbouring sites
*wR*(*F*^2^) = 0.178	H-atom parameters constrained
*S* = 1.03	*w* = 1/[σ^2^(*F*_o_^2^) + (0.0999*P*)^2^ + 2.6202*P*] where *P* = (*F*_o_^2^ + 2*F*_c_^2^)/3
6054 reflections	(Δ/σ)_max_ = 0.001
426 parameters	Δρ_max_ = 0.96 e Å^−^^3^
219 restraints	Δρ_min_ = −0.58 e Å^−^^3^

### Special details

**Table:**

Experimental. The sulfonic group of HSSA dianion is in disorder and has been refined but not satisfactory.
Geometry. All e.s.d.'s (except the e.s.d. in the dihedral angle between two l.s. planes) are estimated using the full covariance matrix. The cell e.s.d.'s are taken into account individually in the estimation of e.s.d.'s in distances, angles and torsion angles; correlations between e.s.d.'s in cell parameters are only used when they are defined by crystal symmetry. An approximate (isotropic) treatment of cell e.s.d.'s is used for estimating e.s.d.'s involving l.s. planes.
Refinement. Refinement of *F*^2^ against ALL reflections. The weighted *R*-factor *wR* and goodness of fit *S* are based on *F*^2^, conventional *R*-factors *R* are based on *F*, with *F* set to zero for negative *F*^2^. The threshold expression of *F*^2^ > σ(*F*^2^) is used only for calculating *R*-factors(gt) *etc*. and is not relevant to the choice of reflections for refinement. *R*-factors based on *F*^2^ are statistically about twice as large as those based on *F*, and *R*-factors based on ALL data will be even larger.

### Fractional atomic coordinates and isotropic or equivalent isotropic displacement parameters (Å^2^)

**Table:**

	*x*	*y*	*z*	*U*_iso_*/*U*_eq_	Occ. (<1)
S3	1.11886 (11)	−0.14488 (10)	0.89754 (7)	0.0681 (4)	0.622 (5)
O6	1.0657 (6)	−0.0994 (5)	0.9516 (3)	0.0971 (16)	0.622 (5)
O7	1.0712 (6)	−0.2372 (4)	0.8826 (3)	0.0973 (17)	0.622 (5)
O8	1.2456 (4)	−0.1450 (6)	0.9124 (4)	0.1026 (19)	0.622 (5)
S3'	1.11886 (11)	−0.14488 (10)	0.89754 (7)	0.0681 (4)	0.378 (5)
O6'	1.0085 (7)	−0.1624 (8)	0.9241 (5)	0.0971 (16)	0.378 (5)
O7'	1.1545 (9)	−0.2352 (6)	0.8675 (5)	0.0973 (17)	0.378 (5)
O8'	1.2068 (8)	−0.1005 (8)	0.9383 (6)	0.1026 (19)	0.378 (5)
Ni1	0.59573 (4)	0.10463 (4)	0.80537 (3)	0.04492 (19)	
S1	0.86708 (13)	0.45268 (11)	0.97477 (9)	0.0867 (5)	
S2	0.42135 (12)	−0.26440 (11)	0.61663 (7)	0.0762 (4)	
O1	0.2056 (7)	0.1245 (5)	0.6004 (4)	0.081 (2)	0.50
H1W	0.1451	0.1176	0.6202	0.122*	0.50
H2W	0.2395	0.1543	0.6298	0.122*	0.50
O2	0.5916 (3)	0.2119 (3)	0.71261 (17)	0.0762 (10)	
H3W	0.5535	0.2426	0.6827	0.114*	
H4W	0.6615	0.2079	0.7065	0.114*	
O3	0.6648 (4)	0.4534 (3)	0.5299 (2)	0.0938 (12)	
H5W	0.6367	0.4593	0.4912	0.141*	
H6W	0.6116	0.4399	0.5528	0.141*	
O4	0.4748 (7)	0.8801 (5)	0.9582 (4)	0.084 (2)	0.50
H7W	0.4223	0.8558	0.9771	0.127*	0.50
H8W	0.4646	0.8593	0.9178	0.127*	0.50
O5	0.6211 (3)	−0.0084 (3)	0.89567 (18)	0.0763 (10)	
H9W	0.5833	−0.0606	0.8975	0.114*	
H10W	0.6840	−0.0261	0.9156	0.114*	
O9	0.7577 (2)	0.0611 (2)	0.79683 (15)	0.0566 (7)	
O10	0.8024 (3)	0.1301 (3)	0.70624 (19)	0.0765 (10)	
O11	1.0017 (3)	0.1011 (3)	0.6679 (2)	0.0915 (13)	
H11	0.9311	0.1125	0.6621	0.137*	
N1	0.6667 (3)	0.2157 (3)	0.86085 (17)	0.0531 (9)	
N2	0.6301 (7)	0.7173 (4)	0.9694 (4)	0.119 (2)	
N3	0.5365 (3)	−0.0069 (3)	0.74554 (18)	0.0545 (9)	
N4	0.0690 (3)	−0.3576 (3)	0.67586 (18)	0.0512 (8)	
C1	0.7555 (4)	0.1994 (3)	0.9076 (2)	0.0577 (11)	
H1	0.7793	0.1363	0.9159	0.069*	
C2	0.8125 (4)	0.2702 (4)	0.9436 (2)	0.0609 (12)	
H2	0.8734	0.2553	0.9756	0.073*	
C3	0.7792 (4)	0.3647 (3)	0.9322 (2)	0.0591 (11)	
C4	0.6873 (5)	0.3829 (4)	0.8847 (3)	0.0680 (13)	
H4	0.6615	0.4454	0.8760	0.082*	
C5	0.6349 (4)	0.3076 (3)	0.8506 (2)	0.0628 (12)	
H5	0.5736	0.3208	0.8184	0.075*	
C6	0.7725 (5)	0.5558 (4)	0.9719 (3)	0.0777 (15)	
C7	0.8082 (7)	0.6385 (5)	0.9465 (3)	0.097 (2)	
H7	0.8803	0.6426	0.9302	0.116*	
C8	0.7340 (9)	0.7170 (5)	0.9456 (4)	0.113 (2)	
H8	0.7579	0.7735	0.9272	0.135*	
C9	0.5986 (7)	0.6361 (6)	0.9958 (4)	0.115 (2)	
H9	0.5272	0.6343	1.0130	0.138*	
C10	0.6680 (6)	0.5523 (5)	0.9989 (4)	0.0945 (19)	
H10	0.6442	0.4967	1.0185	0.113*	
C11	0.4525 (4)	0.0005 (4)	0.6954 (2)	0.0617 (12)	
H11A	0.4178	0.0600	0.6866	0.074*	
C12	0.4151 (4)	−0.0753 (4)	0.6564 (2)	0.0647 (12)	
H12	0.3560	−0.0664	0.6222	0.078*	
C13	0.4639 (4)	−0.1637 (3)	0.6674 (2)	0.0592 (11)	
C14	0.5518 (5)	−0.1722 (4)	0.7173 (3)	0.0743 (14)	
H14	0.5884	−0.2311	0.7258	0.089*	
C15	0.5863 (5)	−0.0938 (4)	0.7548 (3)	0.0733 (14)	
H15	0.6471	−0.1012	0.7882	0.088*	
C16	0.2851 (4)	−0.2967 (3)	0.6419 (2)	0.0548 (10)	
C17	0.2466 (4)	−0.2696 (4)	0.7005 (2)	0.0635 (12)	
H17	0.2928	−0.2305	0.7295	0.076*	
C18	0.1396 (4)	−0.3014 (4)	0.7149 (2)	0.0614 (12)	
H18	0.1148	−0.2827	0.7542	0.074*	
C19	0.1080 (4)	−0.3847 (4)	0.6199 (2)	0.0628 (12)	
H19	0.0610	−0.4249	0.5921	0.075*	
C20	0.2136 (4)	−0.3559 (4)	0.6018 (2)	0.0645 (12)	
H20	0.2369	−0.3763	0.5625	0.077*	
C21	0.8262 (4)	0.0772 (3)	0.7551 (2)	0.0487 (10)	
C22	0.9428 (4)	0.0302 (3)	0.7648 (2)	0.0500 (10)	
C23	0.9732 (4)	−0.0280 (3)	0.8182 (2)	0.0520 (10)	
H23	0.9185	−0.0395	0.8471	0.062*	
C24	1.0833 (4)	−0.0692 (3)	0.8294 (2)	0.0558 (11)	
C25	1.1637 (4)	−0.0526 (4)	0.7859 (3)	0.0692 (13)	
H25	1.2382	−0.0795	0.7934	0.083*	
C26	1.1358 (4)	0.0022 (4)	0.7325 (3)	0.0786 (16)	
H26	1.1907	0.0115	0.7033	0.094*	
C27	1.0251 (4)	0.0452 (4)	0.7209 (2)	0.0638 (12)	

### Atomic displacement parameters (Å^2^)

**Table:**

	*U*^11^	*U*^22^	*U*^33^	*U*^12^	*U*^13^	*U*^23^
S3	0.0600 (7)	0.0674 (8)	0.0720 (8)	0.0162 (6)	−0.0135 (6)	−0.0046 (6)
O6	0.097 (3)	0.101 (4)	0.091 (3)	0.027 (3)	0.001 (3)	0.014 (3)
O7	0.114 (4)	0.080 (3)	0.090 (3)	−0.011 (3)	−0.024 (3)	0.010 (2)
O8	0.074 (3)	0.118 (4)	0.108 (4)	0.007 (3)	−0.026 (3)	0.012 (3)
S3'	0.0600 (7)	0.0674 (8)	0.0720 (8)	0.0162 (6)	−0.0135 (6)	−0.0046 (6)
O6'	0.097 (3)	0.101 (4)	0.091 (3)	0.027 (3)	0.001 (3)	0.014 (3)
O7'	0.114 (4)	0.080 (3)	0.090 (3)	−0.011 (3)	−0.024 (3)	0.010 (2)
O8'	0.074 (3)	0.118 (4)	0.108 (4)	0.007 (3)	−0.026 (3)	0.012 (3)
Ni1	0.0342 (3)	0.0437 (3)	0.0563 (3)	0.0050 (2)	0.0026 (2)	−0.0027 (2)
S1	0.0655 (8)	0.0828 (10)	0.1068 (12)	−0.0091 (7)	−0.0121 (8)	−0.0239 (8)
S2	0.0628 (8)	0.0861 (9)	0.0849 (9)	−0.0234 (7)	0.0313 (7)	−0.0296 (7)
O1	0.090 (5)	0.062 (4)	0.103 (5)	−0.020 (3)	0.061 (4)	−0.011 (3)
O2	0.068 (2)	0.088 (2)	0.073 (2)	0.0191 (18)	0.0125 (17)	0.0177 (19)
O3	0.081 (3)	0.122 (3)	0.076 (2)	0.007 (2)	−0.003 (2)	−0.007 (2)
O4	0.069 (4)	0.059 (4)	0.127 (6)	0.001 (3)	0.020 (4)	0.031 (4)
O5	0.067 (2)	0.072 (2)	0.088 (2)	0.0028 (17)	0.0020 (18)	0.0209 (19)
O9	0.0428 (16)	0.0611 (18)	0.0661 (19)	0.0083 (14)	0.0075 (14)	0.0037 (15)
O10	0.059 (2)	0.089 (2)	0.082 (2)	0.0182 (18)	0.0104 (17)	0.026 (2)
O11	0.068 (2)	0.119 (3)	0.092 (3)	0.016 (2)	0.027 (2)	0.039 (2)
N1	0.0474 (19)	0.053 (2)	0.058 (2)	0.0062 (16)	0.0000 (16)	0.0026 (16)
N2	0.142 (6)	0.066 (4)	0.140 (6)	0.004 (4)	−0.027 (5)	−0.021 (4)
N3	0.0425 (18)	0.057 (2)	0.063 (2)	0.0050 (16)	0.0035 (16)	0.0009 (18)
N4	0.0455 (19)	0.051 (2)	0.057 (2)	−0.0061 (16)	0.0047 (16)	−0.0015 (17)
C1	0.054 (3)	0.053 (3)	0.064 (3)	0.006 (2)	−0.003 (2)	0.008 (2)
C2	0.054 (3)	0.069 (3)	0.057 (3)	0.004 (2)	−0.005 (2)	0.007 (2)
C3	0.056 (3)	0.061 (3)	0.059 (3)	−0.004 (2)	0.004 (2)	−0.009 (2)
C4	0.071 (3)	0.055 (3)	0.073 (3)	0.006 (2)	−0.014 (3)	−0.001 (2)
C5	0.058 (3)	0.057 (3)	0.069 (3)	0.012 (2)	−0.014 (2)	−0.001 (2)
C6	0.081 (4)	0.072 (4)	0.075 (3)	−0.015 (3)	−0.014 (3)	−0.017 (3)
C7	0.125 (6)	0.084 (4)	0.079 (4)	−0.019 (4)	−0.001 (4)	−0.005 (3)
C8	0.148 (8)	0.073 (5)	0.111 (6)	−0.006 (5)	−0.012 (5)	0.001 (4)
C9	0.109 (6)	0.098 (6)	0.135 (7)	0.002 (5)	−0.002 (5)	−0.027 (5)
C10	0.085 (4)	0.072 (4)	0.124 (5)	−0.005 (3)	0.001 (4)	−0.014 (4)
C11	0.050 (2)	0.064 (3)	0.071 (3)	0.008 (2)	0.002 (2)	0.001 (2)
C12	0.055 (3)	0.073 (3)	0.064 (3)	−0.006 (2)	0.000 (2)	−0.005 (2)
C13	0.047 (2)	0.062 (3)	0.071 (3)	−0.010 (2)	0.015 (2)	−0.009 (2)
C14	0.081 (4)	0.051 (3)	0.088 (4)	0.002 (2)	−0.003 (3)	−0.006 (3)
C15	0.074 (3)	0.061 (3)	0.079 (3)	0.013 (3)	−0.018 (3)	−0.002 (3)
C16	0.049 (2)	0.059 (3)	0.057 (3)	−0.003 (2)	0.0089 (19)	−0.002 (2)
C17	0.052 (3)	0.074 (3)	0.066 (3)	−0.020 (2)	0.012 (2)	−0.024 (2)
C18	0.056 (3)	0.071 (3)	0.059 (3)	−0.013 (2)	0.013 (2)	−0.012 (2)
C19	0.060 (3)	0.067 (3)	0.061 (3)	−0.015 (2)	0.006 (2)	−0.011 (2)
C20	0.067 (3)	0.072 (3)	0.057 (3)	−0.017 (2)	0.016 (2)	−0.015 (2)
C21	0.044 (2)	0.045 (2)	0.056 (2)	−0.0004 (17)	−0.0016 (19)	0.0009 (19)
C22	0.041 (2)	0.047 (2)	0.062 (3)	0.0018 (17)	0.0029 (19)	−0.008 (2)
C23	0.047 (2)	0.050 (2)	0.057 (2)	0.0039 (18)	−0.0016 (19)	−0.004 (2)
C24	0.044 (2)	0.051 (2)	0.070 (3)	0.0082 (19)	−0.003 (2)	−0.010 (2)
C25	0.048 (3)	0.069 (3)	0.090 (4)	0.013 (2)	0.005 (3)	−0.007 (3)
C26	0.051 (3)	0.097 (4)	0.091 (4)	0.017 (3)	0.023 (3)	0.006 (3)
C27	0.051 (3)	0.075 (3)	0.066 (3)	0.000 (2)	0.007 (2)	0.008 (2)

### Geometric parameters (Å, °)

**Table:**

S3—O7	1.418 (5)	C2—H2	0.9300
S3—O8	1.450 (5)	C3—C4	1.381 (7)
S3—O6	1.477 (5)	C4—C5	1.365 (7)
S3—C24	1.771 (5)	C4—H4	0.9300
Ni1—O9	1.981 (3)	C5—H5	0.9300
Ni1—N4^i^	2.040 (3)	C6—C7	1.349 (8)
Ni1—N1	2.041 (4)	C6—C10	1.379 (9)
Ni1—N3	2.055 (4)	C7—C8	1.385 (11)
Ni1—O2	2.430 (3)	C7—H7	0.9300
Ni1—O5	2.437 (3)	C8—H8	0.9300
S1—C3	1.759 (5)	C9—C10	1.411 (9)
S1—C6	1.798 (6)	C9—H9	0.9300
S2—C16	1.762 (4)	C10—H10	0.9300
S2—C13	1.789 (5)	C11—C12	1.369 (7)
O1—H1W	0.8504	C11—H11A	0.9300
O1—H2W	0.8000	C12—C13	1.362 (7)
O2—H3W	0.8350	C12—H12	0.9300
O2—H4W	0.8278	C13—C14	1.363 (7)
O3—H5W	0.8338	C14—C15	1.373 (7)
O3—H6W	0.8347	C14—H14	0.9300
O4—H7W	0.8256	C15—H15	0.9300
O4—H8W	0.8821	C16—C20	1.375 (6)
O5—H9W	0.8501	C16—C17	1.390 (6)
O5—H10W	0.8267	C17—C18	1.369 (6)
O9—C21	1.252 (5)	C17—H17	0.9300
O10—C21	1.256 (5)	C18—H18	0.9300
O11—C27	1.348 (6)	C19—C20	1.367 (7)
O11—H11	0.8200	C19—H19	0.9300
N1—C1	1.342 (5)	C20—H20	0.9300
N1—C5	1.343 (6)	C21—C22	1.482 (6)
N2—C9	1.324 (10)	C22—C23	1.384 (6)
N2—C8	1.340 (11)	C22—C27	1.397 (6)
N3—C11	1.337 (6)	C23—C24	1.382 (6)
N3—C15	1.344 (6)	C23—H23	0.9300
N4—C18	1.332 (6)	C24—C25	1.380 (7)
N4—C19	1.344 (6)	C25—C26	1.352 (8)
N4—Ni1^ii^	2.040 (3)	C25—H25	0.9300
C1—C2	1.358 (6)	C26—C27	1.399 (7)
C1—H1	0.9300	C26—H26	0.9300
C2—C3	1.385 (7)		
			
O7—S3—O8	113.4 (4)	C6—C7—H7	121.1
O7—S3—O6	111.9 (4)	C8—C7—H7	121.1
O8—S3—O6	109.2 (4)	N2—C8—C7	124.5 (8)
O7—S3—C24	108.6 (3)	N2—C8—H8	117.7
O8—S3—C24	107.7 (4)	C7—C8—H8	117.7
O6—S3—C24	105.7 (3)	N2—C9—C10	123.2 (8)
O9—Ni1—N4^i^	173.60 (14)	N2—C9—H9	118.4
O9—Ni1—N1	88.00 (13)	C10—C9—H9	118.4
N4^i^—Ni1—N1	90.87 (14)	C6—C10—C9	117.6 (7)
O9—Ni1—N3	87.87 (13)	C6—C10—H10	121.2
N4^i^—Ni1—N3	93.50 (14)	C9—C10—H10	121.2
N1—Ni1—N3	175.23 (14)	N3—C11—C12	123.3 (5)
O9—Ni1—O2	92.99 (12)	N3—C11—H11A	118.4
N4^i^—Ni1—O2	93.24 (13)	C12—C11—H11A	118.4
N1—Ni1—O2	87.06 (14)	C13—C12—C11	120.3 (5)
N3—Ni1—O2	90.79 (14)	C13—C12—H12	119.9
O9—Ni1—O5	80.75 (12)	C11—C12—H12	119.9
N4^i^—Ni1—O5	93.03 (13)	C12—C13—C14	117.4 (4)
N1—Ni1—O5	93.08 (14)	C12—C13—S2	122.2 (4)
N3—Ni1—O5	88.60 (14)	C14—C13—S2	120.3 (4)
O2—Ni1—O5	173.73 (12)	C13—C14—C15	120.0 (5)
C3—S1—C6	103.4 (2)	C13—C14—H14	120.0
C16—S2—C13	102.6 (2)	C15—C14—H14	120.0
H1W—O1—H2W	92.5	N3—C15—C14	123.1 (5)
Ni1—O2—H3W	149.8	N3—C15—H15	118.4
Ni1—O2—H4W	98.5	C14—C15—H15	118.4
H3W—O2—H4W	110.7	C20—C16—C17	117.7 (4)
H5W—O3—H6W	109.9	C20—C16—S2	117.5 (3)
H7W—O4—H8W	106.8	C17—C16—S2	124.8 (3)
Ni1—O5—H9W	124.9	C18—C17—C16	118.9 (4)
Ni1—O5—H10W	126.7	C18—C17—H17	120.6
H9W—O5—H10W	98.2	C16—C17—H17	120.6
C21—O9—Ni1	132.4 (3)	N4—C18—C17	123.8 (4)
C27—O11—H11	109.5	N4—C18—H18	118.1
C1—N1—C5	116.5 (4)	C17—C18—H18	118.1
C1—N1—Ni1	119.9 (3)	N4—C19—C20	123.1 (4)
C5—N1—Ni1	123.5 (3)	N4—C19—H19	118.5
C9—N2—C8	116.5 (7)	C20—C19—H19	118.5
C11—N3—C15	115.9 (4)	C19—C20—C16	119.7 (4)
C11—N3—Ni1	124.7 (3)	C19—C20—H20	120.1
C15—N3—Ni1	119.4 (3)	C16—C20—H20	120.1
C18—N4—C19	116.7 (4)	O9—C21—O10	124.4 (4)
C18—N4—Ni1^ii^	123.2 (3)	O9—C21—C22	117.0 (4)
C19—N4—Ni1^ii^	119.9 (3)	O10—C21—C22	118.6 (4)
N1—C1—C2	123.3 (4)	C23—C22—C27	118.7 (4)
N1—C1—H1	118.3	C23—C22—C21	120.5 (4)
C2—C1—H1	118.3	C27—C22—C21	120.8 (4)
C1—C2—C3	119.6 (4)	C24—C23—C22	121.3 (4)
C1—C2—H2	120.2	C24—C23—H23	119.3
C3—C2—H2	120.2	C22—C23—H23	119.3
C4—C3—C2	118.0 (4)	C25—C24—C23	119.0 (5)
C4—C3—S1	125.2 (4)	C25—C24—S3	120.6 (3)
C2—C3—S1	116.6 (4)	C23—C24—S3	120.3 (4)
C5—C4—C3	118.8 (4)	C26—C25—C24	121.0 (4)
C5—C4—H4	120.6	C26—C25—H25	119.5
C3—C4—H4	120.6	C24—C25—H25	119.5
N1—C5—C4	123.9 (4)	C25—C26—C27	120.5 (5)
N1—C5—H5	118.1	C25—C26—H26	119.8
C4—C5—H5	118.1	C27—C26—H26	119.8
C7—C6—C10	120.3 (7)	O11—C27—C22	121.9 (4)
C7—C6—S1	119.2 (6)	O11—C27—C26	118.7 (5)
C10—C6—S1	120.4 (5)	C22—C27—C26	119.4 (5)
C6—C7—C8	117.9 (8)		
			
N4^i^—Ni1—O9—C21	−172.7 (11)	Ni1—N3—C11—C12	180.0 (4)
N1—Ni1—O9—C21	−92.8 (4)	N3—C11—C12—C13	−0.3 (8)
N3—Ni1—O9—C21	84.9 (4)	C11—C12—C13—C14	−1.4 (7)
O2—Ni1—O9—C21	−5.8 (4)	C11—C12—C13—S2	−178.2 (4)
O5—Ni1—O9—C21	173.8 (4)	C16—S2—C13—C12	−76.2 (4)
O9—Ni1—N1—C1	−45.1 (3)	C16—S2—C13—C14	107.0 (4)
N4^i^—Ni1—N1—C1	128.6 (4)	C12—C13—C14—C15	1.2 (8)
N3—Ni1—N1—C1	−74.9 (18)	S2—C13—C14—C15	178.0 (4)
O2—Ni1—N1—C1	−138.2 (3)	C11—N3—C15—C14	−2.3 (8)
O5—Ni1—N1—C1	35.6 (4)	Ni1—N3—C15—C14	179.7 (5)
O9—Ni1—N1—C5	130.2 (4)	C13—C14—C15—N3	0.7 (9)
N4^i^—Ni1—N1—C5	−56.1 (4)	C13—S2—C16—C20	163.6 (4)
N3—Ni1—N1—C5	100.3 (17)	C13—S2—C16—C17	−18.9 (5)
O2—Ni1—N1—C5	37.1 (4)	C20—C16—C17—C18	−1.1 (8)
O5—Ni1—N1—C5	−149.2 (4)	S2—C16—C17—C18	−178.6 (4)
O9—Ni1—N3—C11	−134.4 (4)	C19—N4—C18—C17	1.0 (7)
N4^i^—Ni1—N3—C11	51.8 (4)	Ni1^ii^—N4—C18—C17	−174.8 (4)
N1—Ni1—N3—C11	−104.5 (17)	C16—C17—C18—N4	0.1 (8)
O2—Ni1—N3—C11	−41.5 (4)	C18—N4—C19—C20	−1.1 (7)
O5—Ni1—N3—C11	144.8 (4)	Ni1^ii^—N4—C19—C20	174.8 (4)
O9—Ni1—N3—C15	43.4 (4)	N4—C19—C20—C16	0.2 (8)
N4^i^—Ni1—N3—C15	−130.3 (4)	C17—C16—C20—C19	0.9 (8)
N1—Ni1—N3—C15	73.3 (18)	S2—C16—C20—C19	178.6 (4)
O2—Ni1—N3—C15	136.4 (4)	Ni1—O9—C21—O10	0.8 (7)
O5—Ni1—N3—C15	−37.4 (4)	Ni1—O9—C21—C22	−179.4 (3)
C5—N1—C1—C2	−0.1 (7)	O9—C21—C22—C23	−0.4 (6)
Ni1—N1—C1—C2	175.4 (4)	O10—C21—C22—C23	179.4 (4)
N1—C1—C2—C3	−0.3 (8)	O9—C21—C22—C27	−179.2 (4)
C1—C2—C3—C4	0.8 (7)	O10—C21—C22—C27	0.6 (6)
C1—C2—C3—S1	−173.6 (4)	C27—C22—C23—C24	1.5 (6)
C6—S1—C3—C4	26.3 (5)	C21—C22—C23—C24	−177.4 (4)
C6—S1—C3—C2	−159.8 (4)	C22—C23—C24—C25	−0.7 (7)
C2—C3—C4—C5	−1.0 (8)	C22—C23—C24—S3	−179.0 (3)
S1—C3—C4—C5	172.8 (4)	O7—S3—C24—C25	−98.3 (5)
C1—N1—C5—C4	−0.1 (8)	O8—S3—C24—C25	24.9 (5)
Ni1—N1—C5—C4	−175.5 (4)	O6—S3—C24—C25	141.5 (5)
C3—C4—C5—N1	0.7 (8)	O7—S3—C24—C23	80.1 (5)
C3—S1—C6—C7	−123.6 (5)	O8—S3—C24—C23	−156.8 (5)
C3—S1—C6—C10	60.5 (5)	O6—S3—C24—C23	−40.1 (5)
C10—C6—C7—C8	−3.4 (9)	C23—C24—C25—C26	−0.8 (7)
S1—C6—C7—C8	−179.3 (5)	S3—C24—C25—C26	177.6 (4)
C9—N2—C8—C7	0.4 (12)	C24—C25—C26—C27	1.4 (9)
C6—C7—C8—N2	1.5 (11)	C23—C22—C27—O11	−179.9 (5)
C8—N2—C9—C10	−0.5 (12)	C21—C22—C27—O11	−1.0 (7)
C7—C6—C10—C9	3.3 (9)	C23—C22—C27—C26	−0.8 (7)
S1—C6—C10—C9	179.2 (5)	C21—C22—C27—C26	178.0 (5)
N2—C9—C10—C6	−1.3 (11)	C25—C26—C27—O11	178.5 (5)
C15—N3—C11—C12	2.1 (7)	C25—C26—C27—C22	−0.6 (8)

Symmetry codes: (i) −*x*+1/2, *y*+1/2, −*z*+3/2; (ii) −*x*+1/2, *y*−1/2, −*z*+3/2.

### Hydrogen-bond geometry (Å, °)

**Table:**

*D*—H···*A*	*D*—H	H···*A*	*D*···*A*	*D*—H···*A*
O11—H11···O10	0.82	1.84	2.535 (5)	142.
O5—H10W···O3^iii^	0.83	1.98	2.797 (5)	170.
O5—H9W···O4^iv^	0.85	2.04	2.721 (8)	136.
O4—H7W···O8^v^	0.83	2.30	2.713 (9)	112.
O3—H6W···O6^vi^	0.83	2.10	2.811 (7)	143.
O3—H5W···O6^vii^	0.83	2.24	2.765 (8)	122.
O2—H4W···O10	0.83	1.95	2.690 (5)	149.
O2—H3W···O7^vi^	0.84	1.87	2.652 (7)	155.
O1—H1W···O11^viii^	0.85	2.03	2.876 (7)	180.

Symmetry codes: (iii) −*x*+3/2, *y*−1/2, −*z*+3/2; (iv) *x*, *y*−1, *z*; (v) *x*−1, *y*+1, *z*; (vi) −*x*+3/2, *y*+1/2, −*z*+3/2; (vii) *x*−1/2, −*y*+1/2, *z*−1/2; (viii) *x*−1, *y*, *z*.
